# Effectiveness of a Pharmacist-Led Cardiovascular Risk Reduction Clinic in Rural Perry County, Alabama

**DOI:** 10.1155/2016/4304761

**Published:** 2016-07-25

**Authors:** Pilar Z. Murphy, Charles Sands, Frances Ford

**Affiliations:** ^1^Department of Pharmacy Practice, Samford University McWhorter School of Pharmacy, 800 Lakeshore Drive, Birmingham, AL 35229, USA; ^2^Sowing Seeds of Hope, 1748 S. Washington Street, Marion, AL 36756, USA

## Abstract

*Background.* The Cardiovascular Risk Reduction Clinic (CRRC) in Perry County, Alabama, provides free pharmacist-led services. Clinic goals include improving health outcomes and reducing cardiovascular risk factors.* Objective.* To investigate the effectiveness of the CRRC in rural Perry County, Alabama. The reduction of the modifiable cardiovascular risk factors, blood pressure and body mass index, was evaluated to measure a decrease from baseline to last clinic date.* Methods.* This retrospective chart review identified 130 patients with at least two blood pressure and BMI measurements from baseline to June 30, 2010. The patients' paper files were used to collect baseline data and most recent measurements, which were recorded on a data collection sheet.* Results.* There was a statistically significant reduction in systolic blood pressure of 4.08 mmHg, 3.25 mmHg reduction in diastolic blood pressure, and 0.42 kg/m^2^ reduction in mean BMI. At their last visit prior to June 30, 2010, 59% of hypertensive patients and 35% of diabetic patients were meeting their blood pressure goals.* Conclusion.* Pharmacist-led management of patients with cardiovascular risk factors significantly reduced blood pressure and allowed more patients to meet their hypertension treatment goals. Despite being modest, reductions in blood pressure and BMI help reduce overall cardiovascular risks.

## 1. Introduction

Perry County, Alabama, is geographically located approximately 75 miles southwest of Birmingham. It is a rural community of about 10,373 residents, with a majority African American population (68%) [1]. The county is plagued with disproportionately higher rates of hypertension, diabetes, and premature mortality, paralleling the trends of extreme poverty and a stagnated economy [1]. In 2010, Perry County was the worst ranked county for health outcomes in the state of Alabama with a ranking of 67 out of 67. By 2013, Perry County had improved in these rankings to 64 out of 67 [1]. According to the most recent chronic disease burden document published by the Alabama Department of Public Health, the public health area including Perry County has hypertension prevalence of 47% [2]. Hypertension is quantitatively the major risk factor for premature cardiovascular disease and is the most common and most important risk factor for stroke [3–5]. Perry County also has one of the highest stroke death rates in America. While the stroke death rate has decreased nationally and in Perry County, reductions in stroke mortality rates in Perry County continue to lag behind the national averages [6, 7]. Perry County also has increased rates of diabetes (20%) and overweight and obesity (78%) [2]. These percentages are much higher than the Alabama prevalence rates of 27.2% for hypertension, 12.2% for diabetes, and 68.1% for overweight and obesity [2]. Overweight and obesity are defined as “abnormal or excessive fat accumulation that may impair health” and are associated with higher rates of hypertension, diabetes, and cardiovascular disease [3, 4].

In 2004, the Cardiovascular Risk Reduction Clinic (CRRC) was established in Perry County as a collaboration between the nonprofit organization Sowing Seeds of Hope, the Perry County Health Department, and Samford University's McWhorter School of Pharmacy. The CRRC provides free pharmacist-led services at the Perry County Health Department. Population surveys indicate that only 52% of adults in the United States with hypertension actually achieve blood pressure target levels [8]. Involvement of pharmacists in hypertension management has been shown to improve blood pressure control [9]. The goals of the clinic include improving health outcomes for hypertension, diabetes, and obesity and the reduction of cardiovascular risk factors for those utilizing the pharmacist-led clinical services.

Patients are encouraged to attend the weekly clinic where they receive free health screenings and medication therapy management. Medication therapy management (MTM) focuses on the assessment and evaluation of the patient's complete medication therapy regimen to optimize and improve therapeutic outcomes for patients [10]. Patients are counseled on cardiovascular disease risk factors and prevention along with proper medication use, drug indications, and medication compliance. When necessary, patients are referred to their primary care physicians for follow-up. Physicians are contacted with recommendations regarding medication changes and needed laboratory tests following an evidence-based approach and clinical guideline recommendations and algorithms. Although the clinic has been in existence since 2004, no data has been published to determine whether CRRC efforts have reduced cardiovascular risk factors.

## 2. Objective

The purpose of this study is to investigate the effectiveness of the pharmacist-led CRRC in rural Perry County, Alabama. More specifically, this retrospective chart review looked at the impact of the CRRC on reducing modifiable cardiovascular risk factors in patients utilizing the clinical services provided. Uncontrolled hypertension and obesity are two of the modifiable risk factors related to cardiovascular disease [12–14] and are addressed with all patients utilizing CRRC services. We evaluated whether patients utilizing the clinic had improved blood pressure and lower body mass indexes since it has been noted that the prevalence of obesity is higher in rural communities [11, 15–18].

## 3. Methods

### 3.1. Clinic Description

The Cardiovascular Risk Reduction Clinic (CRRC), located in the Perry County Health Department, provides free health screenings, disease state management services, and patient counseling to residents of Perry County, Alabama. The CRRC is staffed by a board certified ambulatory care pharmacy specialist, senior pharmacy students completing Advanced Pharmacy Practice Experience (APPE) rotations, licensed registered nurses, and premedical students. While most patients are self-referred, they can be referred to the pharmacist-led clinic by their primary care provider for education and medication management. Data is unavailable to determine the number of patients who have been referred to the clinic but chose not to use the services. Typical patients include elderly patients needing assistance with medication organization and uninsured patients who are in need of help acquiring their prescription medications, therefore presenting with adherence issues.

During the initial visit, a complete medical history is obtained from the patient, a medication list is compiled, and patients receive a health screening including measuring weight, body mass index (BMI), blood glucose, and blood pressure. Patients are also interviewed about their dietary and exercise habits and receive ongoing counseling about proper diet and exercise to help manage their chronic disease states. Participants are provided with assistive materials such as caloric restriction meal plans, information on adopting the Dietary Approaches to Stop Hypertension (DASH) lifestyle eating plan, Healthy Plate for a Healthy Weight placemats with portion control counseling, and information on reducing sodium/salt intake. They are also encouraged to work with CRRC providers to develop measurable and realistic weight loss and exercise goals, along with developing reminders for taking medications, and provided assistance with obtaining medications through patient assistance programs or discounted medications at specific pharmacies to improve access to medications and adherence. During each follow-up visit, the providers assess the patient's medication adherence, weight loss, dietary and exercise habits, and disease state control according to the latest guidelines. If additional information is needed, patients' primary care providers are contacted to access laboratory results and to request information about prescribed medications. Medication recommendations are also forwarded to the primary care providers if CRRC staff believes there is a need to adjust, change, or add an additional medication to optimize therapy for patients not reaching their Joint National Committee on Prevention, Detection, Evaluation, and Treatment of High Blood Pressure (JNC-7) or American Diabetes Association (ADA) blood pressure or blood glucose goals.

### 3.2. Patient Population

A chart review of 253 patients who had an encounter with a pharmacist or pharmacy student in the Perry County Cardiovascular Risk Reduction Clinic was performed by reviewing the paper charts of every individual patient who had been seen in the clinic and had a clinic file. Files were evaluated to identify patients who had at least two clinic visits (a necessity to assess any change from baseline in blood pressure, body mass index, blood glucose, or A1c). Baseline data measurements were collected for the earliest encounter on file. Most recent data measurements for comparison were collected for the latest encounter on record prior to June 30, 2010. The patient's most recent visit must have been at least one month after their initial visit to be included in the study. We decided on an interval between the first and the last visit of at least one month based on guideline recommendations to reevaluate blood pressure within one month when modifying or changing antihypertensive therapies. Baseline demographic data included sex, age, weight, body mass index, blood pressure, blood glucose, and A1c if available. Charts were reviewed for medical history, diagnosis of hypertension including systolic and diastolic blood pressure measurements, diagnosis of diabetes, body mass index (BMI), and weight status (underweight, normal weight, overweight, or obesity). Patients were considered to have hypertension or diabetes if it was self-reported, if it was documented in the medical chart, or if there was current use of antihypertensive or antihyperglycemic medications. Patients were considered to be at a healthy weight, to be overweight, or to be obese based on their BMI measurements related to their height and weight. BMI classifications can be found in Table 1.

All patients included in the study were at least 19 years of age or older and had been seen in the CRRC at least twice during the study period to compare their baseline data to their blood pressure, BMI, and weight. There were 130 patients identified who met this criterion. Patients were excluded from the study if they were under 19 years of age, if they only had one clinic visit, or if their subsequent clinic visit was less than one month after their initial visit. Patients were also excluded if they began utilizing the clinic services after June 30, 2010. Demographic data including race, gender, age, and diagnosis of hypertension or diabetes was gathered on all included patients.

### 3.3. Statistical Analyses

During the time period of the study, every patient chart was reviewed for possible inclusion. All patients meeting the abovementioned criteria were included in the study and a patient list was formulated. Patient information was recorded on data collection sheets (Appendix). The data collection sheet allowed for the assignment of an arbitrary patient number to be used instead of patient names. Each patient received a unique number used specifically for this study. The list of patient names and corresponding numbers was kept separate from the data collection sheets in a locked file cabinet used to store patient files. The primary investigator and senior pharmacy students trained on how to collect the needed information from patient charts were the only ones extracting data for the review. All charts were reviewed on the premises of the CRRC within the Perry County Health Department and were kept confidential in file cabinets currently used to house clinic documents.

We used statistical software SPSS (version 19 for Windows, SPSS Inc.) for data analyses. Clinical value changes from baseline to the last recorded follow-up were analyzed using a matched pair *t*-test. The paired samples were assessed for normal distribution by employing the 2-tailed *t*-tests. Statistical significance was recognized as a *p* value less than or equal to 0.05. This study underwent review and received approval from the Samford University Institutional Review Board (IRB).

## 4. Results

Patient characteristics of the study population are depicted in Table 2. A total of 130 matched enrollees met all inclusion and exclusion criteria. The study population was 69% female, 89% African American, 62% with hypertension, and 35% with both hypertension and diabetes. The average age of the population was 61 years old.

**Figure 1:**
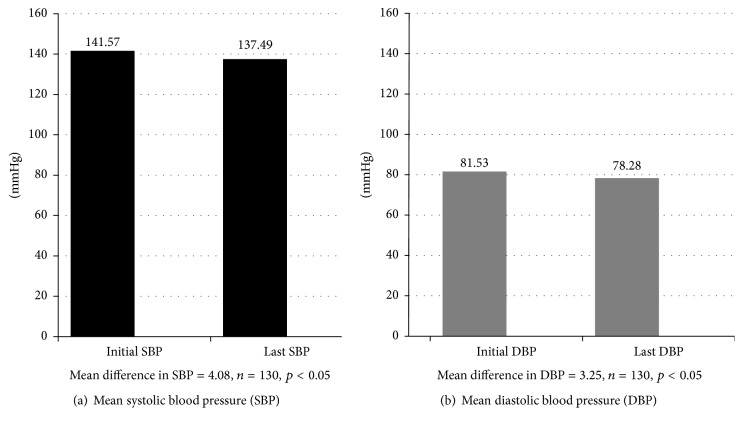
Mean differences for systolic blood and diastolic blood pressure.

**Figure 2:**
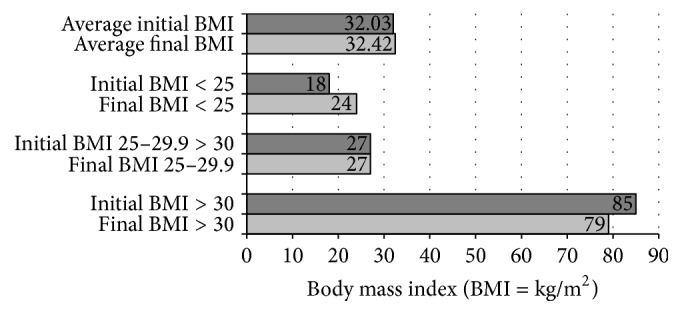
Mean differences in baseline and ending body mass index.

Outcomes data were collected from the charts of all patients who met the inclusion criteria. The matched pair *t*-test was used to measure significant differences in systolic blood pressure and the diastolic blood pressure of the study population during pre- and postintervention periods. The *t*-test assumes that the data is distributed symmetrically. In order to check the violation of the above assumption, we conducted a Wilcoxon Rank Sum test which requires no parametric assumption and showed that the data was distributed symmetrically. At baseline, patients had higher mean systolic/diastolic blood pressure (Figure 1). At the end of the study period, patient groups were successful in reducing systolic/diastolic blood pressure to below 137.49/78.28 mmHg (*p* < 0.05). Mean reduction in systolic blood pressure at the end of the study period was significantly lower from baseline [4.08 (95% confidence interval [CI]: 0.96, 7.19)]. Also, at the end of the study period, the mean diastolic blood pressure significantly changed from baseline [3.25 (95% confidence interval [CI]: 1.31, 5.19)]. There was also a significant reduction in the mean of BMI during the study period. BMI decreased by 0.42 kg/m^2^ (*p* < 0.05) (Figure 2).

## 5. Discussion

We found that the pharmacist-enabled services in the CRRC in Perry County made a significant difference in the blood pressure control of patients. We used the Seventh Report of the Joint National Committee on Prevention, Detection, Evaluation, and Treatment of High Blood Pressure (JNC-7) guidelines to determine whether patients were meeting their blood pressure goals [19]. The JNC-7 guidelines set a hypertension goal for most hypertensive patients at less than 140/90 mmHg and a goal of less than 130/80 for diabetic patients [19]. Upon their initial visit, we had 48% of hypertensive patients meeting the JNC-7 hypertension goal of blood pressure less than 140/90 mmHg. At their last visit prior to June 30, 2010, 59% of the patients were meeting their hypertension goals. This was an improvement of 11%, with an additional 11 patients meeting their goal. Upon their initial visit, 29% of our diabetic patients were meeting their JNC-7 goal of blood pressure of less than 130/80 mmHg. At their last visit, 35% of diabetic patients were meeting their blood pressure goal.

We did see small decreases in both systolic and diastolic blood pressure, showing that clinic efforts are making a difference in blood pressure control. There was a statistically significant drop in SBP of 4.08 mmHg and diastolic blood pressure decreased by an average of 3.25 mmHg. The reduction in SBP moved some patients from being above the 140 mmHg goal to being below it at their last clinic visit. While the average BMI remained constant among patients using the CRRC, six patients moved from the obese category to the overweight category, and six patients moved from the overweight to the normal category. This weight reduction, while being modest, is an improvement in risk since obesity is a modifiable risk factor associated with cardiovascular disease, hypertension, myocardial infarction, and stroke.

During each clinic visit, patient parameters were measured along with the provision of education related to reduction of cardiovascular risk factors, meal planning, weight management, and counseling on medication compliance and utilization. When necessary, patient's providers were contacted with recommendations on changing or augmenting therapy based on the clinical guidelines if patients are not meeting the goal. All of these factors probably have played a role in improving the outcomes of patients using the CRRC.

Centers for Disease Control and Prevention (CDC) statistics show that the stroke death rate in Perry County declined by 37.4% between 1991 and 2010 from 190 to 153 per 100,000 people [7]. While we do not take full credit for this decrease in stroke mortality among patients in Perry County, we do believe that our efforts have had an impact due to increased awareness, patients being educated on the need to seek treatment early, and learning the warning signs of heart attack and stroke. Pharmacy faculty and students provide medication therapy management services to patients utilizing the clinic by making medication review charts and counseling on medication adherence and proper usage, along with providing coaching for lifestyle changes to improve cardiovascular health. Students employ a wallet card provided by the Alabama Department of Public Health which lists the classification of blood pressure, warning signs of a stroke, and the ABCs of heart disease that can be used to talk to patients about aspirin use and smoking cessation. Our model does not propose to be the substitution of physicians in the care of patients with chronic disease states, but rather a division of labor to aid in disease state management and medication therapy management. Our results suggest that pharmacist-enabled clinics do reduce both systolic and diastolic blood pressure among patients and help patients reach their predetermined blood pressure goals. We consider that this clinic model such as ours is a realistic and cost-effective way to provide clinical services to underserved patients, especially those in rural areas.

While many Americans gain weight each year, participants in the CCRC are controlling and maintaining their weight. While only six participants moved from the obese category to the overweight category, the same number of patients moved from being overweight to being in the healthy/normal weight category according to their BMI. Acquiring a normal weight significantly reduces cardiovascular risk factors.

There were limitations to our study. First, there were inconsistencies in documentation in the patient charts. More than 200 charts were reviewed, but, due to documentation errors, omitted information, and incomplete SOAP notes, many patients had to be omitted from the study. Patients were also excluded because they had only one office visit. This loss to follow-up was most concerning to investigators as patients often came to the initial office visit due to symptoms of uncontrolled hypertension or diabetes. These patients may have been referred to their primary care physicians or the emergency room for follow-up but did not return to our clinic for disease state management. Second, there was a lack of a control group for comparison. The lack of a control group prevented us from being able to determine whether clinic efforts were fully responsible for the decreases in blood pressure and weight. Third, we had limited comparison data parameters. We originally wanted to determine whether we had made a difference in diabetes control among patients; however, there was not enough blood glucose data to compare among diabetic patients. Ideally, hemoglobin A1c is a clinical marker used in guidelines and its measurements would be compared to give a more definitive evaluation of blood sugar control.

## 6. Conclusion

The residents of rural Perry County, Alabama, are working to close the gap in cardiovascular health disparities by taking charge of their health and utilizing the CRRC at the Perry County Health Department. The CRRC aims to increase awareness about disease states among patients and to teach patients how to control their risk factors along with taking charge of their comorbid disease state. In summary, the Perry County CRRC model may be an innovative model that is effective in screening patients with hypertension and narrowing the health disparity gap among patients in a rural area. Questions remain as to whether this model is replicable in other areas and whether further improvement in blood pressure control has been realized in patients beyond the study period. The study has also shown the need for more consistent documentation, periodic measurements for hemoglobin A1c and lipids, and an electronic medical record for clinic staff.

## Figures and Tables

**Figure 3:**
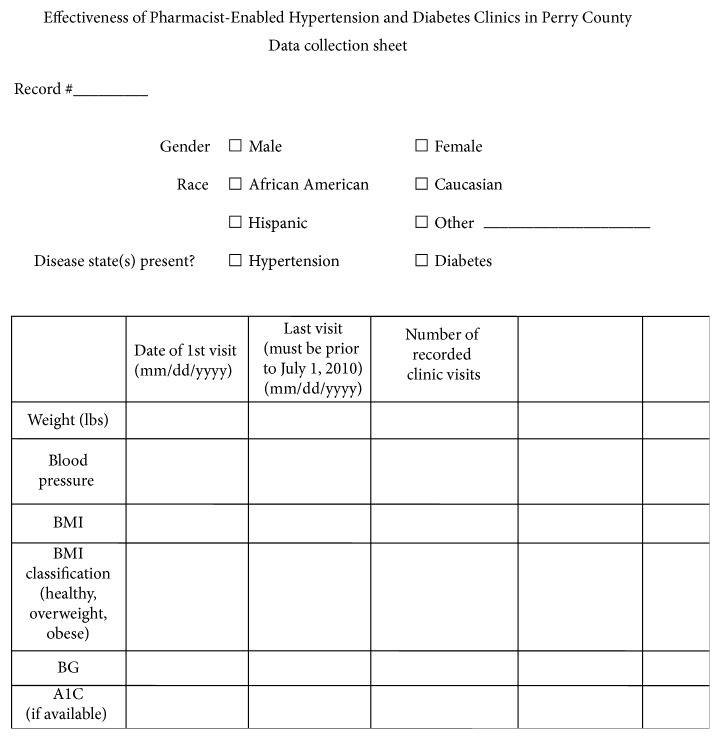


**Table 1:** Classification of overweight and obesity by BMI^*∗*^.

Classification	BMI (kg/m^2^)	Obesity class
Underweight	<18.5	
Normal	18.5–24.9	
Overweight	25.0–29.9	
Obesity	30.0–34.9	I
	35.0–39.9	II
Extreme obesity	40.0+	III

^*∗*^Adapted from the World Health Organization [11].

Body mass index (BMI) is a simple index of weight for height that is commonly used to classify underweight, overweight, and obesity in adults. It is defined as the weight in kilograms divided by the square of the height in meters (kg/m^2^).

**Table 2:** Characteristics of the patients in this study (*n* = 130).

Characteristics	Number	Percentage
*Race*		
African American	116	89
White	14	11

*Sex*		
Female	90	69
Male	40	31

*Age*		
Average (min., max.)	61 (19, 92)	NA

*Reported disease states*		
Hypertension	80	62
Diabetes	3	2
Hypertension & diabetes	46	35
Other/none	1	<1

## Appendix

See Figure 3.
